# Laser-Textured Rubbers with Carbon Nanotube Fillers

**DOI:** 10.3390/polym10101091

**Published:** 2018-10-02

**Authors:** Mariusz Siciński, Ewa Korzeniewska, Mariusz Tomczyk, Ryszard Pawlak, Dariusz Bieliński, Tomasz Gozdek, Karolina Kałuzińska, Maria Walczak

**Affiliations:** 1 Institute of Polymers and Dyes Technology, Faculty of Chemistry, Łódź University of Technology, Stefanowskiego 12/16 Street, 90-924 Łódź, Poland; mariusz.sicinski@p.lodz.pl (M.S.); darisz.bielinski@p.lodz.pl (D.B.); tomasz.gozdek@gmail.com (T.G.); kaluzinska.k@gmail.com (K.K.); 2 Institute of Electrical Engineering Systems, Faculty of Electrical, Electronic, Computer and Control Engineering, Łódź University of Technology, Stefanowskiego 18/22 Street, 90-924 Łódź, Poland; martom@matel.p.lodz.pl (M.T.); ryszard.pawlak@p.lodz.pl (R.P.); mwalczak@matel.p.lodz.pl (M.W.)

**Keywords:** superhydrophobic surface, surface treatment, laser texturing, biomimetics, carbon nanotubes

## Abstract

This paper describes a method of laser ablation for improving the hydrophobic properties of vulcanized rubber. The treatment was tested on acrylonitrile rubber (NBR) and styrene butadiene rubber (SBR) containing carbon nanotubes and soot as fillers. The surface layer of the vulcanizates was modified using a nanosecond-pulsed laser at 1060 nm wavelength. The parameters of the ablation process were congruent, so no chemical changes in the polymeric material were observed. Evaluation of the surface condition of the laser-textured samples was performed using a Leica MZ6 stereoscopic microscope, operating with MultiScan 8.0 image analysis software. The contact angles were determined for all the samples before and after the surface modification process. Following modification of the surface morphology, with the best parameters of laser ablation, the contact angle increased, reaching 147°, which is very close to the threshold of superhydrophobicity (150°). On the basis of the results from several tests, laser ablation with a fiber-pulsed laser can be considered a very useful method for producing rubbers with superhydrophobic surfaces.

## 1. Introduction

**Figure 1:**
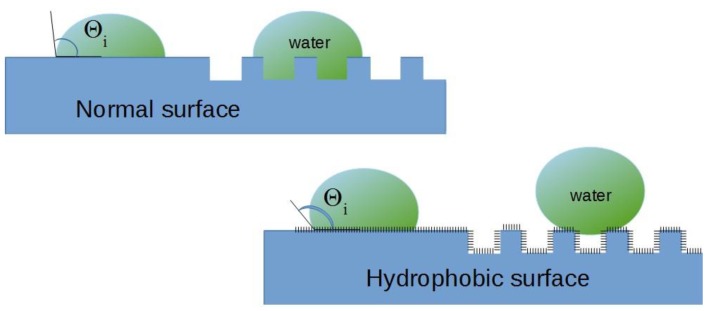
Hierarchy of surface roughness—dependence of contact angle on surface microgeometry.

In recent years, much research has been devoted to the theoretical and experimental aspects of producing surfaces with permanent superhydrophobic effects. A superhydrophobic surface is characterized by a contact angle with water greater than 150°. The superhydrophobic surface exhibits a greater affinity to air than to the liquid that is on it. Neither the chemical composition nor the surface morphology in terms of phase composition and phase distribution are sufficient to achieve a high contact angle. Other important factors include the microroughness and microgeometry of the surface. Figure 1 provides a schematic representation of the hierarchy of surface roughness.

Hierarchical surface structures containing micro and nanoroughness favor the production of superhydrophobic coatings. The wetting properties of such surfaces are determined by the apparent contact angle *θ*′. If water penetrates freely between the rough edges, the apparent wetting angle is calculated from the Wenzel dependence [1]

cos θ* = *r* × cos *θ* (1)

where: *r* ( ≥1) is the ratio of the total surface area between a drop and the surface of a solid body to the projection area of that surface, and *θ* is the contact angle of the surface without microroughness.

In cases where the water (liquid) stops at the uneven peaks (i.e., is partially in contact with the solid body and partially with the air), the apparent contact angle as described by Cassie and Baxter [2] is:

cos θ* = (1 − *φ*_S_) cos *θ*_V_ + *φ*_S_ cos *θ*_S_ (2)

where: (1 − *φ*_S_) and *φ*_S_ are the fractions of the contact surface occupied by air and the solid, respectively, and *θ*_V_ and *θ*_S_ are the contact angles of air and the solid body, respectively. In the case of air cos *θ*_V_ = −1, the dependence on the apparent wetting angle can be written in the form [3]:

cos θ*= –1 + *φ*_S_ (1 + cos *θ*_S_) (3)

In the case of very rough surfaces, *φ*_S_ theoretically tends to 0, and therefore θ* tends to 180°. The surface is then superhydrophobic.

Over the last few decades, the special wetting properties of plants and animal skins have been among the main focuses of research in the emerging field of biomimetics. The surfaces of such materials have a specific hierarchical structure at the micro and nanometer scale, although various chemical substances also play a role in the case of different species. The interactions of these factors give the surfaces excellent physical properties with biological functions, including hydrophobicity. The most famous example is perhaps the self-cleaning surface of a lotus leaf. Hydrophobic wax combined with the characteristic micro and nanostructure of the dips creates a unique superhydrophobic coating. Despite the fact that the natural habitat of the plant is in muddy waters, its leaves remain impeccably clean. Other examples of superhydrophobic surfaces created by nature are butterfly and cicada wings, silver thistle leaves, mosquito eyes, and gecko feet, which thanks to their superhydrophobic properties are also self-cleaning, enabling the nanostructures, which also provide geckos with extraordinary grip, to work efficiently. For practical applications, superhydrophobic surfaces should repel drops under dynamic operating conditions. Many scientists have studied the dynamic effect of droplets on superhydrophobic surfaces. Jung and Bhushan showed that on a surface with changed microgeometry, the transition from a wettable to a non-wettable surface occurs at a critical geometric parameter [4]. Thanks to their understanding of the basic phenomena, researchers have been able to create biomimetic superhydrophobic surfaces for a variety of engineering applications, such as self-cleaning layers [5], in sensors [6], as anti-icing layers in the aviation industry, as anti-pollution layers (e.g., in photovoltaic cells), or as anti-corrosion layers [7,8,9,10].

New polymeric materials containing carbon nanotubes as fillers [11,12], such as acrylonitrile rubber (NBR), styrene butadiene rubber (SBR), and elastomers, require surface treatment to functionalize their properties for a wider range of engineering applications (including hydraulics, pneumatics, machine components, and in the automotive industry). Various surface treatment technologies can be used to obtain specific surface morphologies. Laser ablation of the surface of materials is particularly useful. With proper selection of the laser wavelength (photon energy) and the duration of the laser pulse, changes in the structure of the surface, and chemical changes in the case of polymers, can also be achieved. Methods have been developed to give metallic and semiconducting surfaces hydrophobic surfaces using laser beam ablation with different radiation wavelengths [13,14,15,16]. Due to the possibility it provides of obtaining specific micro and nanostructures, laser beam ablation is used to generate functional surfaces for photovoltaics and in electronics, including elastic electronics, for example, for the production of ordered layers of carbon nanotubes. Laser micromachining is also used to increase adhesion to Teflon in gas phase deposition processes (PVD—physical vacuum deposition) [17] and to shape electroconductive structures on polymer substrates [18,19]. In the case of polymers, methods for producing hydrophobic layers by laser ablation have also been developed; for example, using a CO_2_ laser beam with a wavelength of 9.1–10.64 μm and a pulse duration of 100 ns [20].

The surface modification using plasma is similar to the laser method [21,22,23]. However, the application of plasma can lead to diverse effects, e.g., increasing the wettability of the inner surfaces of polymer pipes [24]. For the hydrophobic layer production, the chemical methods of increasing the polarity of materials surfaces are used [25]. There are also known methods for the creation of foam rubber characterized by a contact angle of 159° used to separate oil from water [26].

The current paper describes a method of laser ablation for improving the hydrophobic properties of vulcanized rubber. The treatment was tested on NBR and SBR containing carbon nanotubes and soot as fillers.

## 2. Materials and Methods

### 2.1. Rubber Samples

Two types of rubber were used for the tests: styrene butadiene rubber (SBR) KER1500 (Dwory S.A., Oświęcim, Poland) and butadiene-acrylonitrile rubber (NBR) PERBUNAN NT 3945 (Lanxess, Cologne, Germany). Multi-wall carbon nanotubes (MWCNT) 95 wt% (Cheaptubes, Cambridgeport, VT, USA) were used as fillers and soot N550 (CB) as absorbers for the laser radiation length. The rubber samples contained 20 weight parts of soot and 5 weight parts of MWCNT per 100 weight parts of rubber. The crosslinking unit used in the rubber vulcanization process consisted of zinc oxide (ZnO), stearin, sulfur, and accelerator TMTD (tetramethylthiuram disulphide)/CBS (N-cyclohexyl-2-benzothiazol-l-sulfenamide). The abbreviations of the mixtures can be found in Table 1.

A laboratory mixer (Brabender Plasticorder, Duisburg, Germany) was used to prepare the mixtures. The components of the mixtures were weighed on a laboratory scale. The rubber was then added into the mixer chamber in order to give it the appropriate degree of plasticity. After this step, stearine, filler residue, TMTD/CBS accelerator, and zinc oxide in the quantities given in Table 2, as well as 1/3 of the filler were added, successively. Finally, sulfur was added as the most important component of the crosslinking unit. The components were dispersed throughout the rubber by mixing. The total preparation time did not exceed 10 min.

Samples for testing were cured at T_0.9_ as determined using an MDR 3000 rheometer (MonTech, Columbia City, IN, USA). The vulcanization time was 10–12 min depending on the composition of the mixture.

### 2.2. Surface Treatment

**Figure 2:**
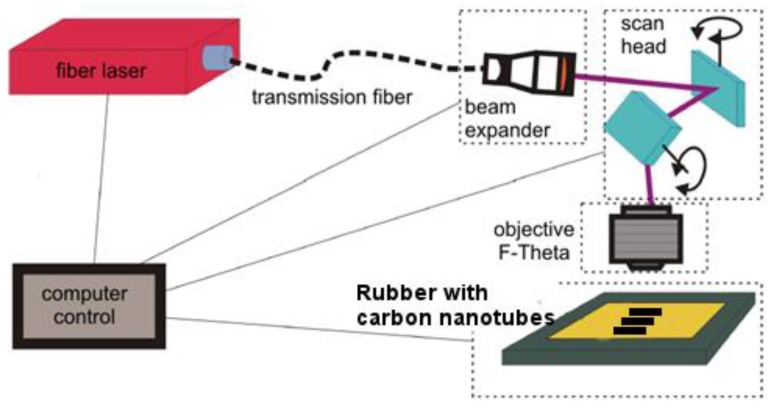
Scheme of the laser system.

Laser modification of the rubber samples was carried out using a single-mode redENERGY G3 SM 20 W fiber laser (SPI, Southampton, UK) equipped with an Xtreme beam scanner (Nutfield Techn. Inc., Hudson, NH, USA). The ablation process was congruent, so no chemical changes occurred in the surface layer of the material. A scheme of the laboratory stand is shown in Figure 2. The beam was transferred from the laser to the scanner via the optical fiber and the expander. The laser modification process was controlled by the Waverunner program, which allowed control of the laser beam parameters, the trajectory speed, and the beam scanning speed. The surfaces of the samples were treated with a 1060 nm laser beam, which was scanned over the surface at a speed in the range of 400–1200 mm/s. The following parameters were varied: pulse energy 25–68 μJ, pulse duration 15–220 ns, and pulse repetition frequency 35–90 kHz. However, on the basis of the scanning speed of the beam and the repetition frequency, a multiple of the impact of a single pulse at a point was determined. The distance between consecutive tracks of the scanning beam (hatching distance) was from 20 to 50 μm during laser texturing.

### 2.3. FTIR Spectroscopic Analysis

The Nicoled 670 spectrophotometer was used for the measurements. As the result of the test, oscillating spectra of samples before and after laser modification were obtained. The analysis of them allowed us to determine the functional groups with which the radiation interacted.

IR spectra were recorded within the wavelength corresponding to the wave vector between 4000 and 450 cm^−1^ using an FTIR Nicolet 6700 FT-IR (Thermo-Scientific, Waltham, MA, USA). The measurement parameters were as follows: 32 scans and resolution 8.

For both types of samples, the butadiene-generated band could be observed at 968 cm^−1^. The styrene band is clearly visible at 698 cm^−1^ for SBR samples. The triple C–N bond-stretching band could be observed at 2238 cm^−1^ for NBR.

**Figure 3:**
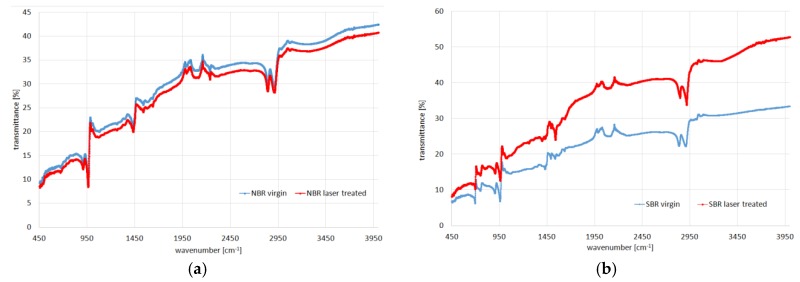
FTIR spectra for (**a**) NBR and (**b**) SBR samples before (blue) and after (red) laser treatment.

FTIR spectra of virgin and modified composites are comparable (Figure 3). They confirmed the lack of chemical changes after the laser treatment. There were transmittance and some intensity differences due to macroscopic surface changes, but any new chemical groups were not visible after the modification.

### 2.4. Evaluation of Laser Modification

**Figure 4:**
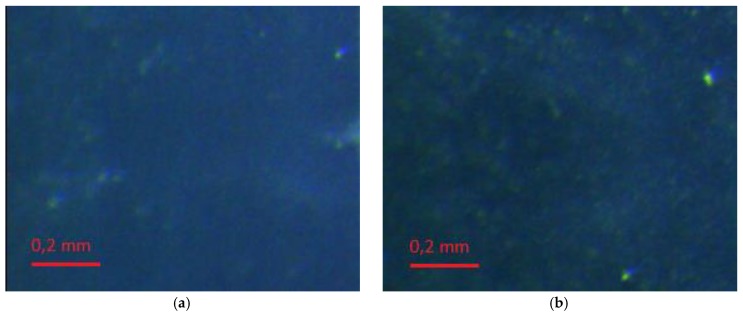

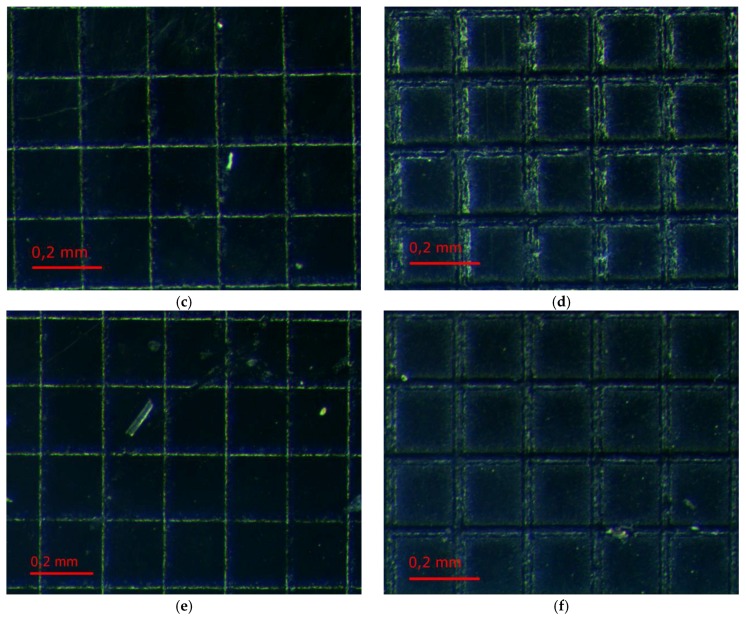
Comparison of rubber surfaces with the addition of MWCNT and CB: before laser treatment (**a**) NBR control sample, and (**b**) SBR control sample; and after laser treatment: (**c**) NBR modified with 2.3 W laser beam power, (**d**) SBR modified with 2.3 W laser beam power, (**e**) NBR modified with 3.3 W laser beam power, and (**f**) SBR modified with 3.3 W laser beam power. Magnification (320×).

Evaluation of the surfaces of the laser textured samples was performed using a Leica MZ6 stereoscopic microscope (Heerbrugg, Switzerland) with MultiScan 8.0 image analysis software (CSS, Poland). Photos of each sample were taken at 100× and 320× magnification. The images are presented in Figure 4. In addition, the surfaces of the modified samples were assessed on the basis of images taken using an AURIGA scanning electron microscope (Zeiss, Göttingen, Germany). The secondary electron signal was used for imaging. The electron beam had 10 keV of energy. The samples had been previously placed in a vacuum deposition chamber to obtain a conductive surface.

The contact angles for all the samples were determined before and after the surface modification process. Droplets with a volume of 2 μL were placed on the surfaces of each sample. Deionized water was used as the measuring liquid. Photos of the drops were made 5 s after they were placed, determining the contact angle by tangents to the external droplet profile on both sides. In each case, five measurements were made and an average value was calculated. Due to the significant effect of microgeometry on the surface roughness of the samples, no free surface energy was determined.

## 3. Results and Discussion

### 3.1. Evaluation of the Rubber Surface Condition after Laser Treatment

Depending on the type of filler, the rubber matrix, and the machining parameters, the surfaces of the laser-treated vulcanizates had different appearances (Figure 4). Beam power was the variable parameter and was one of the most important process parameters. The impact of the laser beam with a given hatching value (0.2 mm) could be observed on the surfaces of all the tested samples. The pictures presented in Figure 4 were made at 320× magnification.

As can be seen, as the power of the laser beam increased the degree, and area of degradation increased in the tracks of the laser beam. A greater degree of degradation with the same hatching parameters and laser beam power was observable for SBR vulcanizates (especially the SBR/MWCTN/CB sample). This was related to the lower thermal stability of the matrix relative to the NBR. Further evidence for this conclusion could be seen in the appearance of the edges of the vulcanizates, which in the case of NBR were sharper. This may have affected the values of the contact angles. During laser beam treatment, a plasma cloud was observed, which indicated that the temperature of the ablated material exceeded 5000 K. It should be noted that such temperatures arose only at a short distance from the treated surface (about 1 mm). The material itself created large temperature gradients (10^7^ K/m) and the temperature rise deep into the material was imperceptible after just a few hundred microns. For such pulse durations, the laser beam was treated as a surface heat source.

**Figure 5:**
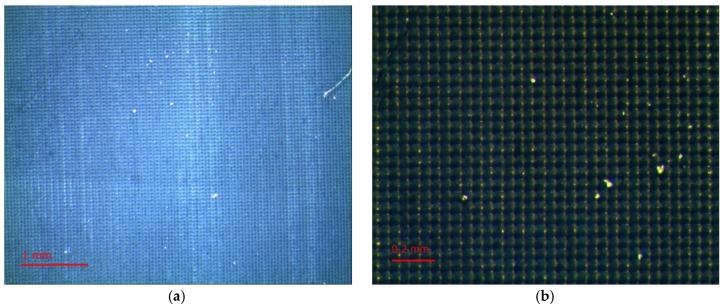
Surface of SBR/MWCNT 50 samples after laser treatment using a beam with a power of 4.5 W and scanning with a hatching distance of 0.05 mm: (**a**) magnification of 100×, and (**b**) magnification of 320×.

**Figure 6:**
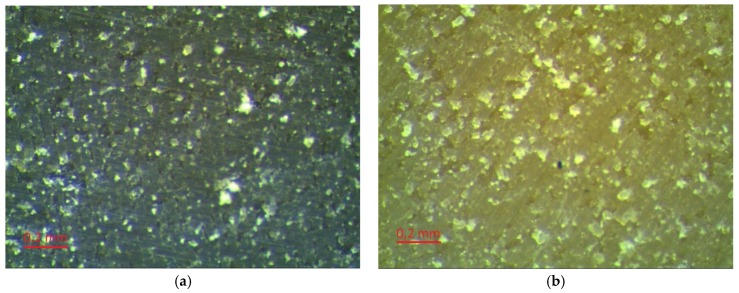

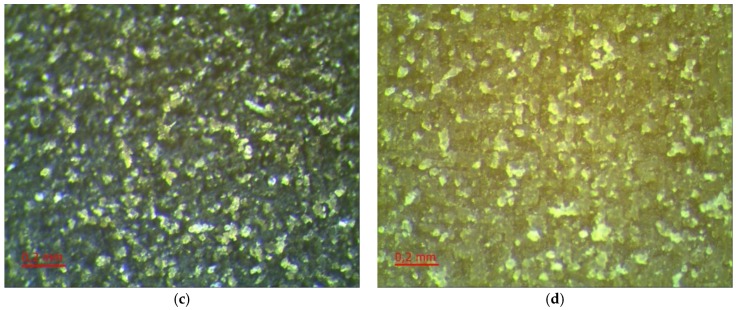
Surface of unfilled samples before laser treatment: (**a**) SBR, and (**b**) NBR; and after laser treatment: (**c**) SBR, and (**d**) NBR (magnification 320×).

As can be concluded from an analysis of the photographs in Figure 5 and Figure 6, the absorption of the laser beam depends on the presence of a carbon filler—CB or MWCNT—which was thermally conductive. Application of a laser beam with the same parameters as for filled samples did not have any effect on the surfaces of the samples without filler, but only caused a slight increase in the temperature of the material. Increasing the power of the beam (exceeding 12 W) led to a violent reaction, resulting in complete thermal degradation of the material, not only on its surface. The unfilled samples subjected to laser treatment (Figure 6) melted and the characteristic traces of laser beam scanning were not even visible on the surface.

### 3.2. Contact Angle of the Rubber Samples after Laser Surface Treatment

**Figure 7:**
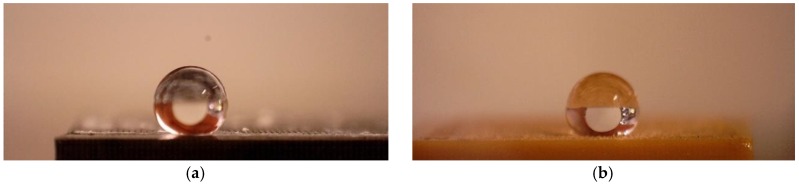
Microscopic pictures of a water droplet on SBR and NBR rubber surfaces after laser modification: (**a**) SBR/MWCNT/50–147°, and (**b**) NBR/MWCNT/CB–126°.

**Figure 8:**
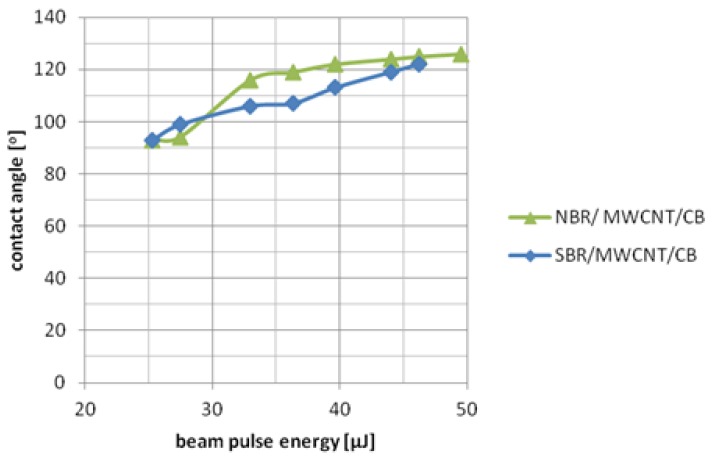
Dependence of contact angle as a function of beam pulse energy for NBR MWCNT/CB and SBR/MWCNT/CB samples.

In order to determine the effects of laser ablation, the surfaces of the tested rubber samples were subjected to a wetting angle test. The results are presented in Table 3, Table 4, Table 5 and Table 6 and in Figure 7 and Figure 8.

Subtle changes in the energy of the laser beam pulse while preserving the same hatching distance did not significantly affect the value of the contact angle. However, increasing the hatching distance by 0.02 mm increased the contact angle by more than 20°. With the same hatching distance, small differences in the energy of the pulse did not affect the changes in contact angles.

As with all technological processes, optimal parameters should be used to achieve the desired effect [27,28]. Analysis of the data contained in Table 4 shows that the optimal conditions for fiber laser treatment of the SBR/MWCNT sample were 0.05 mm hatching with a beam power of 4.5 W. The contact angle reached 147°. This was the closest to the threshold of superhydrophobicity (150°) that was achieved during the tests. In the case of SBR/MWCNT 50, due to its higher thermal conductivity in comparison to vulcanized rubber with soot, higher beam power was used to obtain the intended effect.

All samples that contained in their composition two types of filler were treated using a laser beam with 0.02 mm hatching and beam energy in the range of 25.3–50 μJ. The contact angles of these samples were probably the result of a number of overlapping factors, including the type of filler, the number of fillers, the weight ratios between them, and the morphology of the rubber surface. The degree of dispersion of the filler in the polymer matrix was also important as it affected the absorption of laser radiation. The next important factors to be considered are the hatching distance and pulse energy required for the laser modification.

To better illustrate the effect of laser modification on the contact angle, the results in Table 4 and Table 5 are also presented as a graph in Figure 8. As the laser beam pulse energy increased, the water contact angle for each vulcanite containing a combination of MWCNT and CB increased. In the case of SBR/MWCNT/CB rubber, the dependence of the contact angle on the pulse energy could be approximated by a linear function. For the NBR/MWCNT/CB mixture, an increase in the pulse energy led similarly to an increase in the contact angle of the samples. However, this relationship changed in accordance with the logarithmic function.

Stability of the obtained effect was tested periodically over a period of 3 months. During the research, no changes in the range of contact angles were noticed.

### 3.3. Scanning Electron Microscopy Analysis of the Surfaces of Selected Samples

Scanning electron microscopy (SEM) was used to show the morphology of the rubber samples after fiber laser beam treatment.

**Figure 9:**
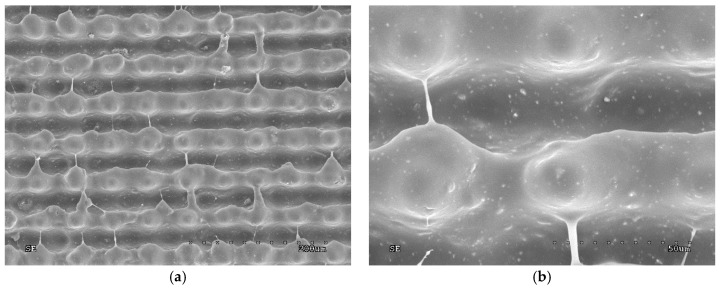

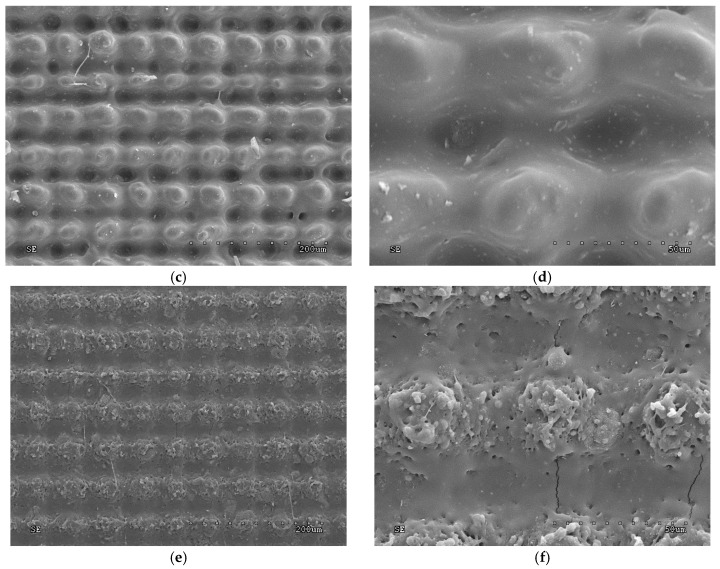
SEM images (magnification 250× and 1000×) of the micro-geometry of SBR/MWCNT/50 samples: (**a**,**b**) 3.5 W beam power, (**c**,**d**) 4 W beam power, and (**e**,**f**) 5 W beam power.

**Figure 10:**
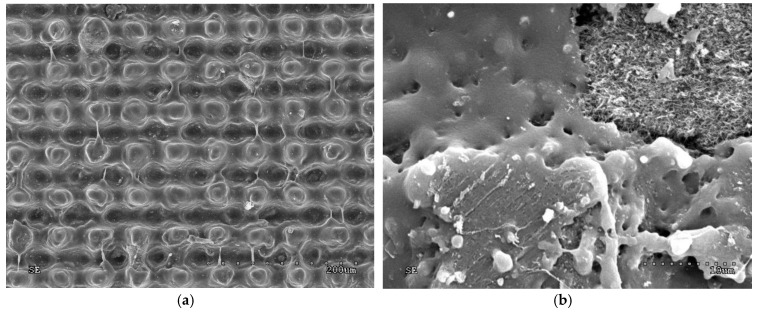
SEM images of the SBR/MWNCT/50 sample for which the largest contact angle (147°) was achieved: magnification (**a**) 250×, and (**b**) 3000×.

The SEM images in Figure 9 and Figure 10 show the hierarchical structure of the sample surfaces. After the laser beam treatment with an optimal average power (4.5 W), agglomerations of MWCNT are clearly visible (Figure 10).

These structures were probably responsible for the very high contact angles, close to those of superhydrophobic materials (147°).

## 4. Summary

In this study, SBR and NBR rubbers containing 5–50 weight parts of nanotubes per 100 weight parts of rubber were subjected to laser ablation to obtain hydrophobic materials. Contact angles with water of up to 147° were achieved on the surfaces, which is a very high value for polymeric materials. The addition of MWCNT as a filler, which increased the thermal conductivity of the material, also improved the hydrophobic properties of the rubbers. Laser ablation generated a hierarchical structure by discovering agglomerates of MWCNT. Doping rubbers with CB also improved the thermal conductivity of the material, although due to the completely different morphology of the filler particles, a hierarchical structure was not produced. In the case of rubber filled with structural CB, the tendency of the filler to form an internal structure prevailed over the agglomeration of its particles. Hence, a hierarchical structure was not generated, as was the case with MWCNT.

Our goal was to increase the contact angle. Unfortunately, a side effect of the treatment of the surface layer of rubbers with a laser beam is that it may have caused thermal degradation and oxidation of the polymer. This had a counterproductive effect where the contact angle relative to water decreased. The optimal modification conditions should be determined to create a favorable microstructure that most effectively increased the contact angle.

## Figures and Tables

**Table 1:** Abbreviations of compound names with their explanations.

Mixture	Explanations
SBR_0_	SBR without filler
SBR/CB	SBR with the addition of soot
SBR/MWCNT	SBR with the addition of carbon nanotubes
SBR/MWCNT/CB	SBR with the addition of carbon nanotubes and soot
NBR_0_	NBR without filler
NBR/MWCNT/CB	NBR with the addition of carbon nanotubes and soot

**Table 2:** Compositions of rubber mixtures (in weight parts).

Composition Abbreviation	Rubber	CB	MWCNT	ZnO	Stearin	CBS	Sulphur
SBR/MWCNT/CB	100	20	5	5	1	2	2
SBR/CB 50	100	50	-	5	1	2	2
SBR/MWCNT 50	100	-	50	5	1	2	2
NBR/MWCN/CB	100	20	5	5	1	2	2

**Table 3:** Change in the contact angle for SBR/CB samples as a result of laser modification of their surfaces.

SBR/CB 50	Power of the Beam (W)	Beam Pulse Energy (mJ)	Hatching (mm)	Contact Angle (°)		
				Before Laser Treatment	After Laser Treatment	Resulting Difference
1.	3.0	0.0330	0.02	99	107	8
2.	3.5	0.0385	0.02		107	8
3.	3.0	0.0330	0.04		128	29
4.	3.5	0.0385	0.04		132	33

**Table 4:** Changes in wetting angle of SBR/MWCNT samples as a result of laser modification of their surfaces.

SBR/MWCNT 50	Power of the Beam (W)	Beam Pulse Energy (mJ)	Hatching (mm)	Contact Angle (°)		
				Before Laser Treatment	After Laser Treatment	Resulting Difference
1.	3.5	0.0385	0.04	91	140	49
2.	4.0	0.044	0.05		133	42
3.	4.5	0.0495	0.05		147	56
4.	5.0	0.055	0.05		144	53

**Table 5:** Changes in the contact angle as a function of beam power and hatching for NBR/MWCNT/CB samples.

NBR/MWCNT/CB	Beam Power (W)	Beam Pulse Energy (µJ)	Hatching (mm)	Contact Angle (°)		
				Before Laser Modification	After Laser Modification	Resulting Difference
1.	2.3	25.3	0.02	69	93	24
2.	2.5	27.5			94	25
3.	3.0	33			116	47
4.	3.3	36.3			119	50
5.	3.6	39.6			122	53
6.	4.0	44			124	55
7.	4.2	46.2			125	56
8.	4.5	49.5			126	57

**Table 6:** Changes in the contact angle as a function of beam power and hatching for SBR/MWCNT/CB samples.

SBR/MWCNT/CB 5:20	Beam Power (W)	Beam Pulse Energy (µJ)	Hatching (mm)	Contact Angle (°)		
				Before Laser Modification	After Laser Modification	The Resulting Difference
1.	2.3	25.3	0.02	71	93	22
2.	2.5	27.5			99	28
3.	3.0	33			106	35
4.	3.3	36.3			107	36
5.	3.6	39.6			113	42
6.	4.0	44			119	48
7.	4.2	46.2			122	51
8.	4.5	49.5			126	55
9.	5.0	55			129	58
